# Room temperature structure of human IgG4-Fc from crystals analysed *in situ*

**DOI:** 10.1016/j.molimm.2016.11.021

**Published:** 2017-01

**Authors:** Anna M. Davies, Theo Rispens, Pleuni Ooijevaar-de Heer, Rob C. Aalberse, Brian J. Sutton

**Affiliations:** a King’s College London, Randall Division of Cell and Molecular Biophysics, New Hunt’s House, London SE1 1UL, United Kingdom; b Medical Research Council & Asthma UK Centre in Allergic Mechanisms of Asthma, London, United Kingdom; c Sanquin Research, Amsterdam 1066 CX, The Netherlands; d University of Amsterdam, Academic Medical Centre Landsteiner Laboratory, The Netherlands

**Keywords:** IgG4, Antibody, Immunoglobulin, X-ray crystallography

## Abstract

The Fc region of IgG antibodies (Cγ2 and Cγ3 domains) is responsible for effector functions such as antibody-dependent cell-mediated cytotoxicity and phagocytosis, through engagement with Fcγ receptors, although the ability to elicit these functions differs between the four human IgG subclasses. A key determinant of Fcγ receptor interactions is the FG loop in the Cγ2 domain. High resolution cryogenic IgG4-Fc crystal structures have revealed a unique conformation for this loop, which could contribute to the particular biological properties of this subclass. To further explore the conformation of the IgG4 Cγ2 FG loop at near-physiological temperature, we solved a 2.7 Å resolution room temperature structure of recombinant human IgG4-Fc from crystals analysed *in situ*. The Cγ2 FG loop in one chain differs from the cryogenic structure, and adopts the conserved conformation found in IgG1-Fc; however, this conformation participates in extensive crystal packing interactions. On the other hand, at room temperature, and free from any crystal packing interactions, the Cγ2 FG loop in the other chain adopts the conformation previously observed in the cryogenic IgG4-Fc structures, despite both conformations being accessible. The room temperature human IgG4-Fc structure thus provides a more complete and physiologically relevant description of the conformation of this functionally critical Cγ2 FG loop.

## Highlights

• Room temperature structure of human IgG4-Fc solved from crystals analysed *in situ*.
• Structure reveals changes in crystal packing at different temperatures.
• Structure reveals physiologically relevant conformation of a key Fcγ receptor binding loop.

## 1 Introduction

IgG effector functions, such as antibody-dependent cell-mediated cytotoxicity, antibody-dependent cellular phagocytosis and complement activation, are mediated by the antibody Fc region (Cγ2 and Cγ3 domains). The IgG4 subclass binds certain Fcγ receptors with lower affinity than IgG1 and IgG3 (Bruhns et al., 2009), and does not activate complement (van der Zee et al., 1986). Antibody determinants that influence the affinity for Fcγ receptors include sequence variation in the Cγ2 domain and hinge region, and the composition of the oligosaccharide moiety attached to the Cγ2 domain (Canfield and Morrison, 1991, Shields et al., 2002).

The Cγ2 domain FG loop (residues 325–331) plays a crucial role in the interaction with Fcγ receptors, in which Pro329 from the FG loop forms a hydrophobic “proline sandwich” interaction with two tryptophan residues from the receptor (Sondermann et al., 2000), and is also involved in the interaction between IgG1/3 and C1q (Canfield and Morrison, 1991, Tao et al., 1991, Tao et al., 1993, Idusogie et al., 2000). While the conformation of the Cγ2 domain FG loop is conserved in IgG1, high resolution cryogenic crystal structures of IgG4-Fc revealed a different, unique conformation for the Cγ2 FG loop, which would disrupt the interaction with Fcγ receptors (Davies et al., 2014b). Subsequent cryogenic crystal structures of IgG4-Fc (Davies et al., 2014a) and intact IgG4 (Scapin et al., 2015) revealed that the IgG4 Cγ2 FG loop could also adopt the conserved IgG1-like conformation. However, the role of the unique loop conformation in modulating the biological properties of IgG4, and whether one, or both, conformations could be adopted at physiological temperature, and in solution, remains unclear.

Using a technique to collect data from crystals *in situ* (Axford et al., 2015), we solved a 2.7 Å resolution room temperature (RT) structure of recombinant human IgG4-Fc. The RT IgG4-Fc structure reveals conformational diversity in the Cγ2 FG loop. In contrast to the cryogenic structure, the FG loop adopts the IgG1-like conformation in one Cγ2 domain, with substantial changes to the crystal packing interactions at the higher temperature which would preclude the unique conformation due to steric clashes. On the other hand, the FG loop from the other Cγ2 domain is able to adopt either conformation − in fact it adopts the unique, IgG4-like conformation at room temperature, a conformation that would disrupt the interaction with Fcγ receptors.

## 2 Materials and methods

### 2.1 Protein production and crystallisation

Recombinant, glycosylated human IgG4-Fc was produced and crystals were grown as described previously (Davies et al., 2014b), with the following modification: a Greiner Bio-One CrystalQuick™ X plate was set up using a reservoir volume of 20 μL, and drops comprising 0.5 μL protein (3 mg/mL) and 0.5 μL reservoir. Crystals typically started to appear after one day.

### 2.2 Data collection, structure determination and refinement

**Fig. 1:**
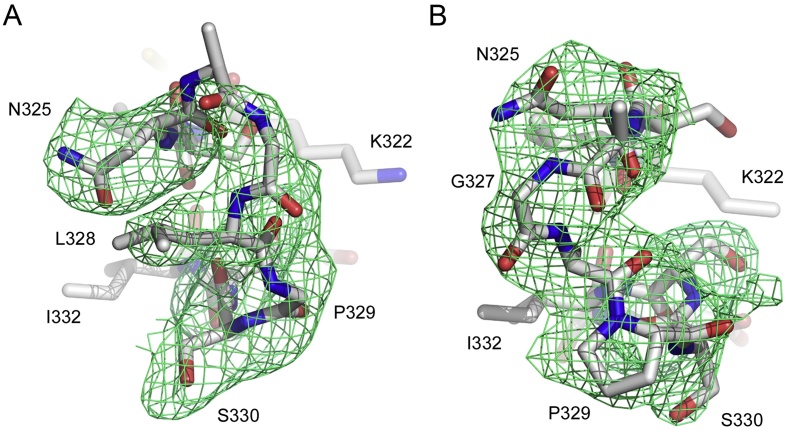
Electron density for the Cγ2 domain FG loop. (A) Cγ2 FG loop from chain A. (B) Cγ2 FG loop from chain B. F_o_-F_c_ maps are shown, contoured at 2.5σ. Residues 325–331 were omitted from the model prior to refinement.

Data were collected at room temperature (293 K) at beamline I03 at the Diamond Light Source (Harwell, UK) from crystals *in situ*. Small wedges (typically 3°−6°) of data were collected from different crystals, or spatially distinct regions from a single crystal, using an oscillation per image of 0.2°. For multiple datasets collected from a single crystal, the oscillation start angle for each dataset was incremented by 2°. Over 200 partial datasets from 48 isomorphous crystals were collected in this manner. Integration was performed with XDS (Kabsch, 2010) within the *xia2* package (Winter, 2010) and further processing was carried out using POINTLESS (Evans, 2011), SORTMTZ, AIMLESS (Evans and Murshudov, 2013) and TRUNCATE (French and Wilson, 1978) from the CCP4 suite (Winn et al., 2011). Only the first 10 images (2° of data) from each partial dataset that had been successfully integrated with XDS, with Batch R_merge_ values of 40% or less, were typically used for scaling, with 129 runs of data finally included. The structure was solved by molecular replacement with PHASER (McCoy et al., 2007) using protein atoms from PDB: 4C54 as a search model, with residues 325–331 omitted from the model. Refinement was performed with PHENIX (Adams et al., 2010), using the “Optimize X-ray/stereochemistry weight” and “Optimize X-ray/ADP weight” options, and manual model building was performed with *Coot* (Emsley et al., 2010). For both chains of the asymmetric unit, the Cγ2 domain FG loop conformation was validated by inspection of 2F_o_-F_c_ and F_o_-F_c_ electron density maps following refinement with residues 325–331 omitted from the model (Fig. 1). Structure quality was assessed with MolProbity (Chen et al., 2010) within PHENIX. Data processing and refinement statistics are presented in Table 1. Interfaces were analysed with PISA (Krissinel and Henrick, 2007) and figures were produced with PyMOL (The PyMOL Molecular Graphics System, Version 1.1r1, Schrödinger, LLC).

## 3 Results and discussion

### 3.1 Overall structure

**Fig. 2:**
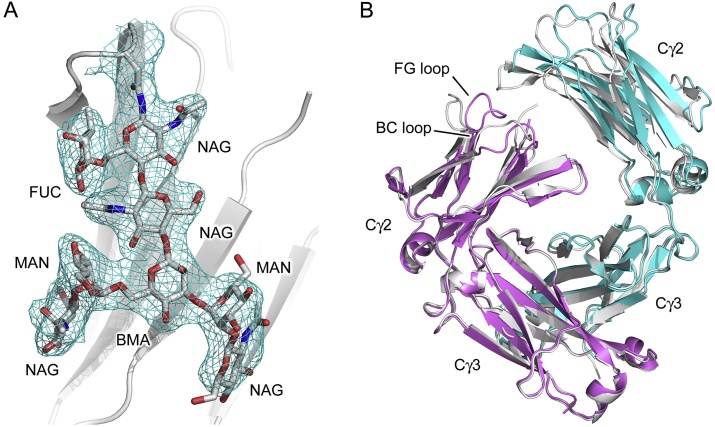
Overall structure of human IgG4-Fc solved at room temperature from crystals *in situ*. (A) Electron density for the oligosaccharide moiety from chain A. A 2F_o_-F_c_ map is shown, contoured at 1σ. The oligosaccharide residues are labelled as follows: BMA, β-d-mannose; NAG, N-acetylglucosamine; MAN; α-d-mannose, FUC, α-l-fucose. (B) Overall structure of human IgG4-Fc. Chains A and B for the room temperature structure are colored blue and purple, respectively. For comparison, the cryogenic IgG4-Fc structure (Davies et al., 2014b) is shown in gray. The disposition of the Cγ2 domain from chain A differs when the two structures are superposed (RMSD 0.36 Å for chain B Cγ3 domain Cα atoms). The positions of the Cγ2 BC and FG loops are indicated for chain B. (For interpretation of the references to colour in this figure legend, the reader is referred to the web version of this article.)

The asymmetric unit of the room temperature (RT) recombinant human IgG4-Fc crystal structure solved from crystals *in situ* contains one Fc molecule, comprising two chains (A and B). Residues Gly236-Ser444 and Gly237-Ser444 were built for chains A and B, respectively. A heptasaccharide core, covalently linked to Asn297 in the Cγ2 domain, was modelled for each chain. Each oligosaccharide moiety additionally contains a fucose residue attached to the first N-acetylglucosamine residue. The quality of the electron density map is illustrated for the oligosaccharide moiety from chain A in Fig. 2A.

The RT structure belongs to the same crystal form (space group *P* 2_1_ 2_1_ 2_1_) previously reported for the cryogenic recombinant IgG4-Fc crystal structure (Davies et al., 2014b), and the overall domain topology is comparable. However, the Cγ2 domains adopt a slightly more “open” conformation at room temperature *i.e.* they are further apart from one another compared with their position in the cryogenic structure (Fig. 2B). For example, the Cα atoms for Val323 are 34.8 Å and 36.0 Å apart in the cryogenic and RT structures, respectively.

Despite belonging to the same crystal form, the *b* and *c* unit cell dimensions in the RT structure (*b* = 81.93 Å, *c* = 103.88 Å) are ∼3 Å and 6 Å longer, respectively, than those in the cryogenic recombinant IgG4-Fc structure (*b* = 78.97 Å, *c* = 97.88 Å). The longer unit cell dimensions at room temperature are mostly attributed to a conformational difference in the Cγ2 FG loop in chain B.

### 3.2 Crystal packing interactions for the Cγ2 domain of chain A

**Fig. 3:**
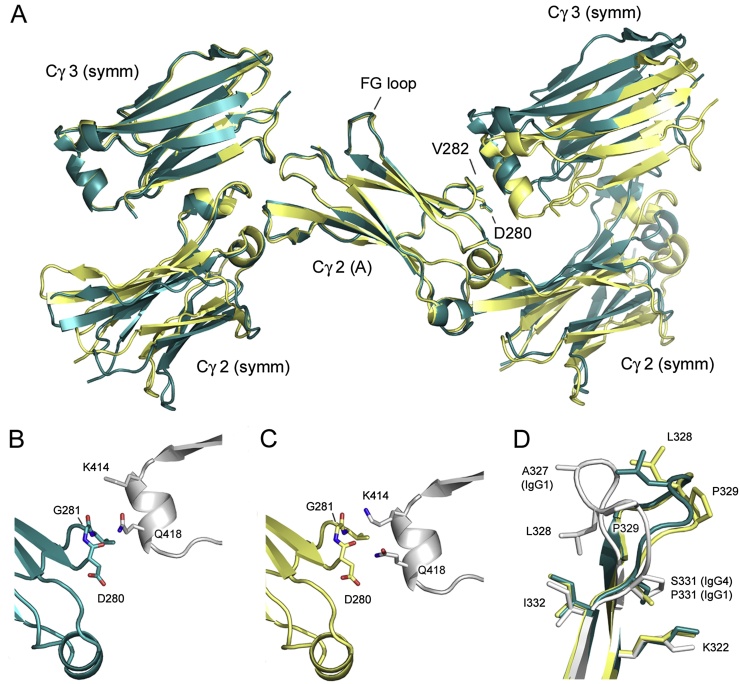
Crystal packing and Cγ2 domain FG loop conformation (chain A). (A) With the exception of residues Asp280-Val282, crystal packing interactions in the room temperature (RT) (blue) and cryogenic (yellow) (Davies et al., 2014b) IgG4-Fc structures, which do not involve the FG loop, are similar. (B) In the RT structure (blue) Gln418 from the Cγ3 domain of a symmetry-related molecule (gray) packs against residues Asp280, Gly281 and Val282. (C) In the cryogenic structure (yellow), Lys414 packs against Gly281, and Gln418 forms a hydrogen bond with the Asp280 carbonyl atom. (D) In chain A of the RT (blue) and cryogenic (yellow) structures, the Cγ2 FG loop from chain A adopts a different conformation to the conserved conformation found in IgG1 (gray) (Ferrara et al., 2011). Figures (A–C) were generated after superposition on the Cγ2 domain from chain A. (For interpretation of the references to colour in this figure legend, the reader is referred to the web version of this article.)

With the exception of residues Asp280-Val282, crystal packing interactions for the Cγ2 domain from chain A are similar in both RT and cryogenic structures (Fig. 3A). At room temperature, Gln418 from the Cγ3 domain of a symmetry-related molecule packs against Asp280-Val282, forming a 96 Å^2^ interface (105 Å^2^ if disordered atoms are included) (Fig. 3B). On the other hand, crystal packing in the cryogenic structure is such that the relative position of the helix containing Gln418 is altered; instead, Lys414 packs against Gly281, and Gln418 forms a hydrogen bond with the Asp280 carbonyl atom, creating a larger interface of 132 Å^2^ (Fig. 3C).

### 3.3 At room temperature, the Cγ2 domain FG loop adopts the unique, IgG4-like conformation in chain A

The Cγ2 FG loop conformation is conserved in both unbound human IgG1-Fc and receptor-bound IgG1-Fc crystal structures (Davies and Sutton, 2015). However, the cryogenic crystal structure for human IgG4-Fc revealed a different Cγ2 FG loop conformation, which would disrupt the interaction with Fcγ receptors (Fig. 3D) (Davies et al., 2014b). We now report that at room temperature, the Cγ2 FG loop adopts the unique, IgG4-like conformation (Figs.1A and 3D) as seen in the cryogenic structure.

At room temperature, average *B* factors for chain A chain Cγ2 FG loop residues are ∼1.8 fold higher than the Cγ2 domain average, a value comparable to the cryogenic structure average (∼1.7 fold higher), but in contrast to the cryogenic structure, the Lys326 and Pro329 side chain atoms are disordered at room temperature. Although the IgG1-like conformation is not precluded in chain A, we did not observe electron density to suggest that the loop samples both IgG1-like and IgG4-like conformations.

### 3.4 Interplay between crystal packing and Cγ2 domain FG loop conformation in chain B

**Fig. 4:**
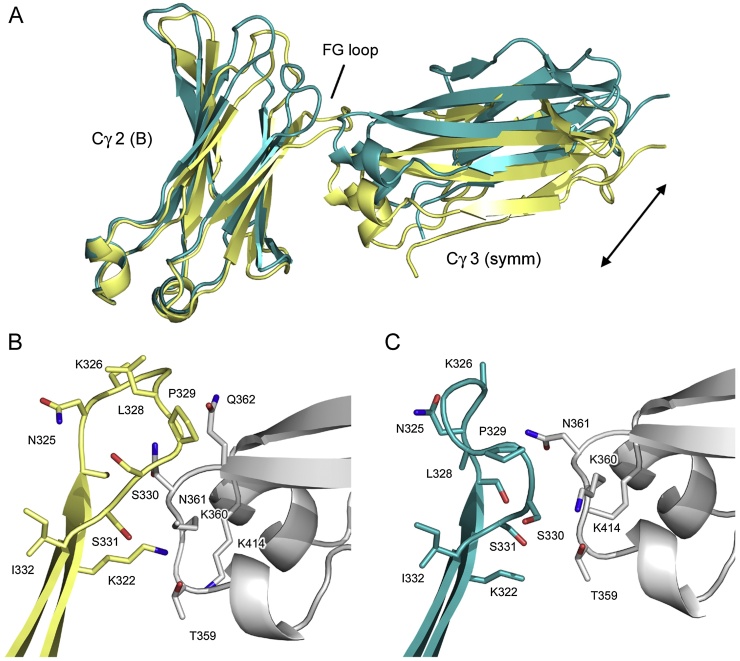
Crystal packing and Cγ2 domain FG loop conformation (chain B). (A) The Cγ2 domain FG loop from chain B adopts a different conformation in the room temperature (RT) (blue) and cryogenic (yellow) (Davies et al., 2014b) IgG4-Fc structures. In the RT structure, the Cγ2 FG loop adopts the conserved conformation found in IgG1. In the cryogenic structure, the unique, IgG4-like conformation would clash with the Cγ3 domain from a symmetry-related molecule (Cγ3-symm), and the crystal packing alters to accommodate this conformation, with a reduction in the *c* unit cell dimension, the direction of which is indicated by an arrow. (B) In the cryogenic structure, the Cγ2 FG loop (yellow) adopts the unique, IgG4-like conformation and forms a 288 Å^2^ interface with a symmetry-related molecule (gray). (C) In the RT structure, the IgG1-like Cγ2 FG loop (blue) forms a 161 Å^2^ interface with a symmetry-related molecule (gray). Figure (A) was generated after superposition of the Cγ3 domains from the room temperature and cryogenic structures. Figures (B) and (C) were generated after superposition on the Cγ2 domain from chain B. (For interpretation of the references to colour in this figure legend, the reader is referred to the web version of this article.)

Substantial crystal packing interactions occur between the Cγ2 domain FG loop from chain B and the Cγ3 domain from a symmetry-related molecule in the *P* 2_1_ 2_1_ 2_1_ crystal form (Fig. 4A). In the cryogenic structure, the nature of these interactions are such that either IgG1-like or IgG4-like conformations are accessible, but the loop adopts the unique, IgG4-like conformation (Fig. 4B). On the other hand, and in contrast to the cryogenic structure, the FG loop adopts the conserved IgG1-like conformation at room temperature (Fig. 4C); steric clashes with the symmetry-related molecule preclude the IgG4-like conformation (Fig. 4A).

At room temperature, with the FG loop in an IgG1-like conformation, a 161 Å^2^ interface (182 Å^2^ if disordered atoms are modelled) with the symmetry-related molecule includes: three hydrogen bonds; contacts between main chain atoms from residues 325 and 328–330, and main chain and side chain atoms from the symmetry-related Cγ3 domain; packing of the Ser330 side chain against Thr359 and Lys360 main chain and side chain atoms (Fig. 4C). In the cryogenic structure, with the FG loop in an IgG4-like conformation, an even larger, 288 Å^2^ interface (295 Å^2^ if disordered atoms are modelled) includes: five hydrogen bonds; contacts between main atoms from residues 325 and 327–330, and main chain and side chain atoms from the symmetry-related Cγ3 domain; packing of the Ser330 side chain against Thr359 and Lys360 main chain atoms, and Pro329 against Gln362 (Fig. 4B).

Strikingly, as the contact area with the symmetry-related molecule increases upon cryocooling, the crystal lattice contracts, reducing *c* by 6 Å, accommodating the different loop conformation (Fig. 4A).

### 3.5 Cγ2 domain BC loop conformation

The Cγ2 domain BC loop (residues 264–273) also plays a role in the interaction between IgG and Fcγ receptors (Davies and Sutton, 2015, Kiyoshi et al., 2015, Oganesyan et al., 2015), and IgG and C1q (Idusogie et al., 2000). This loop conformation is conserved in unbound IgG1-Fc, and the majority of IgG1-Fc/Fcγ receptor complex structures, but the cryogenic IgG4-Fc structure (*P* 2_1_ 2_1_ 2_1_ crystal form) revealed a different conformation, due to isomerisation of Pro271 (Davies and Sutton, 2015). Crystal structures of IgG1-Fc in complex with FcγRI later demonstrated that the IgG1 Cγ2 BC loop could also adopt this different conformation, which facilitates the formation of a hydrogen bond with the receptor; however, the Cγ2 FG loop conformation was conserved (IgG1-like) (Kiyoshi et al., 2015, Oganesyan et al., 2015). Together with the deglycosylated IgG4-Fc structure (Davies et al., 2014a), in which an IgG4-like FG loop conformation and IgG1-like BC loop conformation were observed in the same Cγ2 domain, the IgG1-Fc/FcγRI complex structures suggest that the Cγ2 BC and FG loop conformations are independent of one another (Davies and Sutton, 2015).

**Fig. 5:**
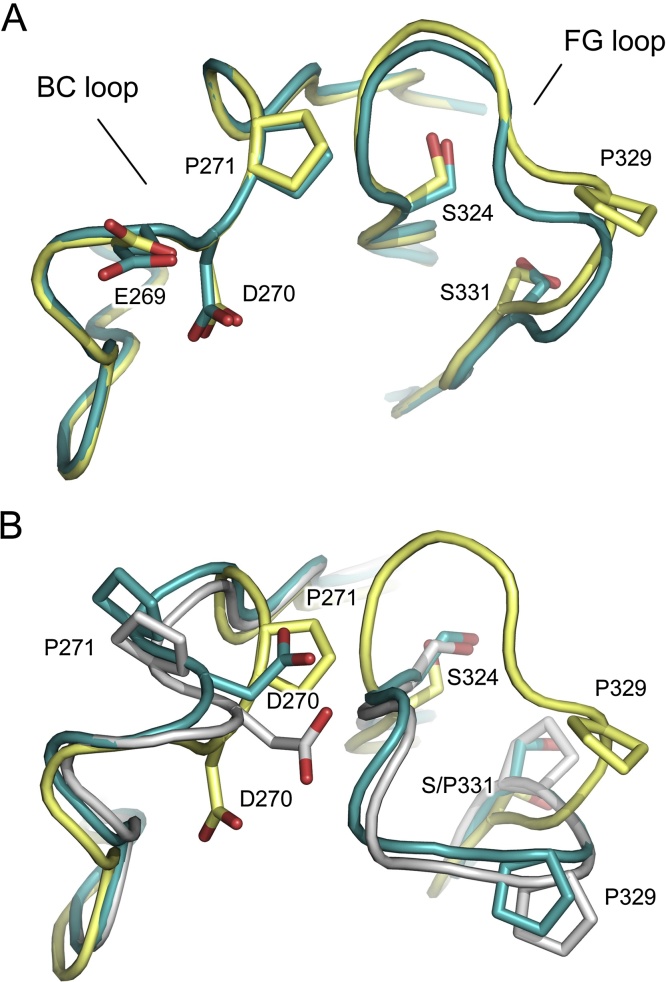
Cγ2 domain BC loop conformation. (A) The Cγ2 domain BC and FG loops from chain A adopt similar conformations in the room temperature (RT) (blue) and cryogenic (yellow) IgG4-Fc structures (Davies et al., 2014b). (B) The Cγ2 domain BC and FG loop conformations from chain B of the RT structure (blue) differ from those in the cryogenic structure (yellow), and are more akin to the conformations typically observed in IgG1-Fc (gray) (Ferrara et al., 2011). (For interpretation of the references to colour in this figure legend, the reader is referred to the web version of this article.)

In chain A of the RT IgG4-Fc structure, the Cγ2 BC (and FG) loop adopts the same conformation observed in the cryogenic structure (Fig. 5A). On the other hand, the Cγ2 BC (and FG) loop from chain B adopts the conformation which is conserved in the majority of IgG1-Fc structures (Fig. 5B). Thus, while the Cγ2 BC and FG loops have been observed to independently adopt conserved and non-conserved conformations within the same domain, we do not see any evidence for this in the RT IgG4-Fc structure.

### 3.6 Implications of the Cγ2 domain FG loop conformation

The ability of the IgG4 Cγ2 domain FG loop to adopt two different conformations, one IgG1-like and one IgG4-like, as observed in chain B of the RT and cryogenic IgG4-Fc structures respectively, was previously seen in the four independent chains of the cryogenic structure of deglycosylated human IgG4-Fc, which crystallised in a different crystal form (*P* 6 2 2) (Davies et al., 2014a). However, in these chains, both loop conformations are involved in crystal packing interactions (with an average area of ∼147 Å^2^). More recently, a crystal structure of intact IgG4, solved under cryogenic conditions, revealed an IgG1-like Cγ2 FG loop conformation in one chain, in the absence of crystal packing interactions (Scapin et al., 2015).

By contrast, in the *P* 2_1_ 2_1_ 2_1_ crystal form, chain A forms only minor interactions with a symmetry-related molecule. Under cryogenic conditions, Leu328 from the IgG4-like Cγ2 FG loop forms an interface of ∼35 Å^2^ with Gln311 from a symmetry-related molecule; this interaction is ∼4 Å^2^ at room temperature, attributed to disorder of the Gln311 side chain and differences in overall packing (although, the interaction area is ∼16 Å^2^ if the disordered side chain is modelled). These values suggest that the Cγ2 FG loop conformation for this chain more closely reflects the situation in solution.

Importantly, the RT IgG4-Fc crystal structure, in which either IgG1-like or IgG4-like conformations are accessible in chain A, reveals that the Cγ2 FG does not sample multiple conformations at room temperature. In fact, the unique, IgG4-like Cγ2 FG loop conformation is adopted, which would disrupt the interaction with Fcγ receptors, in contrast to the conserved, IgG1-like conformation, which would engage Fcγ receptors through the “proline sandwich” interaction.

The recent crystal structure of intact IgG4 (Scapin et al., 2015) revealed an unusual position for one of the Cγ2 domains, which was rotated by ∼120°, exposing the carbohydrate moiety covalently attached to Asn297. In the FG loop from this domain, torsion angles for Lys326 (ψ) (103°), Gly327 (φ) (132°) and Ser331 (φ) (−133°) are comparable to the range of torsion angle values for the unique IgG4-like conformation in the cryogenic and RT IgG4-Fc structures [Lys326 (ψ), 102 to 125°; Gly327 (φ) 77 to 128°; Ser331(φ), −143 to −160°], which differ from the range of typical torsion angle values found in high resolution IgG1-Fc structures [Lys326 (ψ), −3 to −41°; Ala327 (φ), −66 to −103°; Pro331 (φ), −41 to −71°]. By contrast, the Ser330 (φ) torsion angle (−111°) is more akin to the range of values for Ala330 (φ) in IgG1-Fc (-115 to −159°), which differs from the range of values for Ser330 (φ) when the unique IgG4-like conformation is adopted (-61 to −81°). When the rotated Cγ2 domain is superposed in turn on Cγ2 domains from IgG1-Fc and IgG4-Fc structures, the overall position of Pro329 from the FG loop is closer to the position of Pro329 in IgG1-Fc. However, when the FG loop alone is compared, the positions of Lys326 and Gly327 main chain atoms are more similar to those for the unique IgG4-like conformation. The IgG4-like conformation that would disrupt the “proline sandwich” interaction with the receptor is precluded in this rotated domain, as the FG loop packs against the other Cγ2 domain. However, this raises an intriguing possibility that if this conformation were adopted, it could modulate the position of the rotated Cγ2 domain, and influence overall IgG4 structure.

Although both IgG1-like and IgG4-like Cγ2 FG loop conformations are accessible in chain A, the unique IgG4-like conformation is adopted at both temperatures when crystal packing interactions do not pose any steric restrictions; the room temperature structure could reflect the loop conformation in solution, although we cannot rule out that the loop is more dynamic under these conditions and adopts different conformations. Indeed, both IgG1-like and IgG4-like Cγ2 FG loop conformations bury a similar surface area against the rest of the Cγ2 domain. The IgG1-like loop conformation is clearly accessible, but the influence exerted by the local environment, such as the packing interactions with a symmetry-related molecule in chain B, on the energy barrier between the two conformations, and their relative energies, requires further exploration.

## 4 Conclusions

In summary, we solved the room temperature structure of recombinant human IgG4-Fc at 2.7 Å resolution from crystals *in situ*. Comparison of this structure with the recombinant human IgG4-Fc structure previously solved in the same space group under cryogenic conditions revealed that the Cγ2 FG loop from one chain (chain B) adopts the conserved IgG1-like conformation at room temperature, and the unique IgG4-like conformation under cryogenic conditions. In this chain, the two loop conformations are associated with substantial changes in crystal packing interactions at the two different temperatures. However, our structure also demonstrates that when either conformation is accessible, the Cγ2 FG loop from the other chain (chain A) does not sample multiple conformations, and adopts the unique, IgG4-like conformation at near-physiological temperature.

## Accession number

Coordinates and structure factors have been deposited in the Protein Data Bank with accession number PDB: 5LG1.

## Figures and Tables

**Table 1:** Data collection, processing and refinement statistics.

Data collection	
Number of crystals used	48
Number of datasets collected	>200 partial datasets
Temperature (K)	293
Data processing	
Space group	*P* 2_1_2_1_2_1_
Unit cell dimensions	
*a*, *b*, *c* (Å)	73.29, 81.93, 103.88
Resolution (Å)	48.35−2.70 (2.83−2.70)^a^
Completeness (%)	99.9 (99.9)^a^
Multiplicity	8.7 (8.9)^a^
Mean ((*I*)/σ(*I*))	12.2 (2.0)^a^
CC_1/2_	0.992 (0.433)^a^
R_merge_ (%)	17.1 (288.4)^a^
R_pim_ (%)	6.0 (98.9) a
Wilson *B* factor (Å^2^)	77.7
Refinement	
R_work_/R_free_ (%)^b^	17.53/23.13
RMSD	
Bond lengths (Å)	0.003
Bond angles (°)	0.540
Coordinate error (Å)	0.39
No. of atoms	
Protein^c^	3270
Oligosaccharide A/B	99/99
Solvent	20
Ave. *B* factor (Å^2^)	
Protein: Cγ2 A/B	74.9/115.3
Protein: Cγ3 A/B	72.3/69.8
Oligosaccharide A/B	86.8/143.3
Solvent	60.6
Ramachandran plot^d^	
Favoured (%)	98.6
Allowed (%)	100

a Values in parentheses are for the highest resolution shell.

b R_free_ set comprises 5% reflections.

c Includes alternative conformations.

d Ramachandran plot generated by MolProbity (Chen et al., 2010).
